# Rolling Element Bearing Fault Diagnosis under Impulsive Noise Environment Based on Cyclic Correntropy Spectrum

**DOI:** 10.3390/e21010050

**Published:** 2019-01-10

**Authors:** Xuejun Zhao, Yong Qin, Changbo He, Limin Jia, Linlin Kou

**Affiliations:** 1 State Key Laboratory of Rail Traffic Control and Safety, Beijing Jiaotong University, Beijing 100044, China; 2 School of Traffic and Transportation, Beijing Jiaotong University, Beijing 100044, China; 3 National Engineering Laboratory for System Safety and Operation Assurance of Urban Rail Transit, Guangzhou 510000, China; 4 Beijing Research Center of Urban Traffic Information Sensing and Service Technologies, Beijing Jiaotong University, Beijing 100044, China; 5 School of Mechanical Engineering, Dalian University of Technology, Dalian 116024, China

**Keywords:** fault diagnosis, cyclostationary, kernel method, correntropy, impulsive noise

## Abstract

Rolling element bearings are widely used in various industrial machines. Fault diagnosis of rolling element bearings is a necessary tool to prevent any unexpected accidents and improve industrial efficiency. Although proved to be a powerful method in detecting the resonance band excited by faults, the spectral kurtosis (SK) exposes an obvious weakness in the case of impulsive background noise. To well process the bearing fault signal in the presence of impulsive noise, this paper proposes a fault diagnosis method based on the cyclic correntropy (CCE) function and its spectrum. Furthermore, an important parameter of CCE function, namely kernel size, is analyzed to emphasize its critical influence on the fault diagnosis performance. Finally, comparisons with the SK-based Fast Kurtogram are conducted to highlight the superiority of the proposed method. The experimental results show that the proposed method not only largely suppresses the impulsive noise, but also has a robust self-adaptation ability. The application of the proposed method is validated on a simulated signal and real data, including rolling element bearing data of a train axle.

## 1. Introduction

Rolling element bearings are one of the most widely used mechanical components in various industrial machines, such as gearboxes, railway axles, and turbines. Their health states tend to degrade due to repeating rotations under harsh working conditions. Fault diagnosis of rolling element bearings is essential to prevent machine breakdown and ensure production efficiency. Therefore, research related to bearing degradation prognostics and fault diagnosis has recently attracted much attention [1,2,3].

The narrowband-based amplitude demodulation of a vibration signal enables the extraction of more detailed information about the fault signature. The procedure of this method uses a band-pass filter to retain one of the resonant frequency bands. Hilbert transform is then employed to demodulate the signal preprocessed by the band-pass filter and to construct a squared envelope on the filtered signal [4]. At last, the squared envelope spectrum is utilized to identify bearing fault frequencies. Thus, the key of the narrowband-based amplitude demodulation is to find a more informative resonant frequency band for Hilbert demodulation. Much effort has been made to address this issue. Spectral kurtosis (SK), also known as Kurtogram [5,6], proposed by Antoni, is one of the most pioneering works in the field. Kurtogram uses kurtosis to quantify an analytical signal obtained by a band-pass filter as well as Hilbert transform so as to characterize the impulsiveness of bearing fault signals. Furthermore, an SK-based algorithm named Fast Kurtogram [7] was developed to make it a tool with potential online industrial applications. Fast Kurtogram returns the complex envelopes of the signal in selected frequency bands with an arborescent multi-rate filter-bank structure, thus reducing the computation complexity. This SK-based algorithm has inspired many related works on the fault diagnosis of rotating machines [8,9,10].

However, vibration signals of bearings contain impulsive noise in some industrial situations, which bring challenges to further applications of this technology. Moreover, the Kurtogram algorithm was found to be sensitive to impulsive noise and this can sometimes lead to incorrect interpretations [11].

A number of improved methods have been proposed in order to analyze and solve this problem, such as the smoothness index-guided approach [12], the sparsity index-guided approach [13], and the Protrugram analysis [14]. Among these works, the Protrugram analysis proposed by Barszcz and JabLoński has gained the most attention. The Protrugram uses the kurtosis of the envelope spectrum instead of the kurtosis of the filtered time signal to characterize the bearing fault. Despite its unique contribution, the Protrugram has the dual limitations of the spectral Kurtogram and it raises the problem of selecting an optimal mathematical expression in its definition among many possible choices [15].

Motivated by ideas from the field of thermodynamics, a new concept named Infogram was proposed [15], which involves measuring the squared envelope, the squared envelope spectrum, and their average as estimators to select the fault frequency band. It has been proved that the method is able to detect the impulsive and cyclostationary signature in both time and frequency domains. This work stresses the importance of the suppression of impulsive noise; meanwhile, it has the potential to deal with impulsive noise based on cyclostationary modeling.

Cyclostationarity is a property that characterizes stochastic processes whose statistical properties periodically vary with respect to some generic variable [16]. This generality makes cyclostationarity nicely suited to rotating machine signals because of hidden periodicities in its structure. Based on this cyclostationary modeling theory, cyclic spectral analysis, also known as spectral correlation (SC) analysis, has been developed as a reliable tool for fault diagnosis of rolling element bearings [17,18].

As a second-order cyclostationary component extraction indicator, SC analysis has become popular in the field of machine diagnostics. SC analysis [17] can simultaneously describe the carrier and modulation for bearing fault diagnosis by means of a bi-spectral map. Here, the carrier refers to the spectral band of frequencies and the modulation refers to the cyclic frequency. In other words, the cyclic frequency describes the periodicity of the fault impacts, while the spectral band of frequencies corresponds to the resonance characteristic of the mechanical system. Thus, the fault frequency distribution of the signal can be identified over the resonance frequency bands. However, SC may be extremely costly to compute in some situations. To solve this problem, alternative estimators have been proposed, such as averaged cyclic periodogram [17], cyclic modulation spectrum [19], and fast estimator of SC [20]. These estimators greatly relieve SC from computation burdens and contribute to SC analysis for bearing fault diagnosis.

Recently, a new cyclostationary analysis technology named cyclic correntropy (CCE) analysis has emerged to suppress impulsive noise [21,22,23]. CCE is a kernel-based similarity measure of cyclostationary modeling signals. Related research has shown that it has a good suppression performance when dealing with the binary phase-shift keying signal under an impulsive noise environment [21,23]. However, to the best of our knowledge, only several works related to CCE analysis have been published in the field of communication and its applications need to be developed and reinforced. Furthermore, a CCE analysis of vibration signal processing has never been explored before. Therefore, for the first time, this paper introduces CCE into the analysis of bearing vibration signal processing. Furthermore, this paper also investigates the influence of kernel size on the CCE performance, which is critical in the CCE analysis. Thus, this is proven to be a promising fault diagnosis method for bearings as well as other rotating machinery in the presence of impulsive noise.

The rest of this paper is outlined as follows. In Section 2, the fundamentals of correntropy function and the cyclic spectral analysis of rotating machine vibration signals are reviewed. In Section 3, cyclostationary analysis based on the correntropy function and its spectrum for bearing fault diagnosis is proposed. In Section 4, simulated and real bearing fault signals including a vibration dataset acquired from industrial railway axle bearings are used to verify the effectiveness of the proposed method. Moreover, the SK-based Fast Kurtogram method is conducted to highlight the superiority of the proposed method. Conclusions are drawn in the final section.

The intended contributions of this study can be summarized as follows:

1. A cyclostationary analysis method based on the correntropy function is introduced into the fault diagnosis of the rolling element bearing under an impulsive noise environment.
2. The kernel size of the correntropy function is investigated to find out its influence on the rolling element bearing fault diagnosis.
3. The diagnosis performance of the proposed method is compared with the Fast Kurtogram method using train axle bearing data to verify its suitability.

## 2. Fundamentals of Correntropy Function and Cyclic Spectral Analysis

### 2.1. Correntropy Function

Correntropy function can be regarded a similarity measure based on kernel function. The definition of correntropy function is defined as follows [24]:

Let $\left\{x_{t},t\in T\right\}$ be a stochastic process with T being an index set and $x_{t}\in R^{d}$. The correntropy function $V(t_{1},t_{2})$ is defined as a function from T∗T into $R^{+}$ given by Equation (1):

(1) $$V(t_{1},t_{2})=E[\kappa (x_{t_{1}}-x_{t_{2}})]$$

where E[·] denotes the mathematical expectation over the stochastic process $x_{t}$. $\kappa (x_{t_{1}}-x_{t_{2}})$ corresponds to the positive-definite function which satisfies the Mercer condition [25]. The Gaussian kernel is usually chosen as the Mercer kernel function due to its smoothness and strict positive-definiteness [26]. The kernel function is shown in Equation (2):

(2) $$\kappa (x-y)=\frac{1}{\sqrt{2\pi }\sigma }\exp-(\frac{||x-y|{|}^{2}}{2\sigma^{2}})$$

where σ is the kernel size. The correntropy function is dependent upon the kernel size and it is selected according to certain applications [27].

By applying an extension of the Taylor series to the correntropy function, Equation (1) can be rewritten as follows [24]:

(3) $$V(t_{1},t_{2})=\frac{1}{\sqrt{2\pi }\sigma }\sum_{n=0}^{\infty }\frac{{\left(-1\right)}^{n}}{2^{n}\sigma^{2n}n!}E[||x_{t_{1}}-x_{t_{2}}|{|}^{2n}]$$

which involves all the even-order moments of the random variable $\left||x_{t_{1}}-x_{t_{2}}|\right|$. Specifically, the term corresponding to n=1 in Equation (3) is proportional to:

(4) $$E\left[||x_{t_{1}}|{|}^{2}\right]+E\left[||x_{t_{2}}|{|}^{2}\right]-2E[<x_{t_{1}},x_{t_{2}}>]=\sigma_{x_{t_{1}}}^{2}+\sigma_{x_{t2}}^{2}-2R_{x}(t_{1},t_{2})$$

where $R_{x}(t_{1},t_{2})$ is the covariance function of the random process. Thus, the information provided by the traditional correlation function is included within the new function. It can be seen that the correntropy function is a second-order statistic of the mapped feature space data. Besides, the correntropy function incorporates higher-order moments of the random variable $\left||x_{t_{1}}-x_{t_{2}}|\right|$ by adjusting the values of the kernel size.

Thanks to these attractive properties, the correntropy function has been widely applied in machine learning and signal processing. Among those applications, the suppression performance of non-Gaussian noise has been highly researched [28,29,30], in which the Gaussian kernel function plays an important part. The superiority of the Gaussian kernel function mainly includes two points: transforming values of outliers into zero and extracting higher statistical moments. These two merits enable the correntropy function to handle signals corrupted by non-Gaussian noise, especially impulsive noise.

### 2.2. Cyclic Spectral Analysis

A cyclostationary process is a stochastic process that exhibits some hidden periodicities in its structure [16]. Denote a bearing fault signal as $x(t_{n})$ and its stream as x[n], where $t_{n}=n/F_{s},n=0,1,\ldots ,L-1$ indicate time instants with sampling frequency $F_{s}$. Assume that the bearing fault signal is cyclostationary on the second order, which indicates that the instantaneous autocorrelation function is periodic with period *T* [20]:

(5) $$R_{x}\left(t_{n},\tau \right)=Ε\left\{x\left(t_{n}\right)x{\left(t_{n}-\tau \right)}^{*}\right\}=R_{x}\left(t_{n}+T,\tau \right)$$

where E{⋅} refers to the ensemble average operator; $\tau =m/F_{s}$; and ∗ is the complex conjugate operator.

One important tool for the cyclic spectral analysis of bearing signals is SC analysis, which is obtained in the form of a bi-spectral map, thus reflecting the whole picture of spectral frequency f and cyclic frequency α in the signal. Based on Equation (5), SC is defined as follows [20]:

(6) $$S_{x}\left(\alpha ,f\right)=\underset{N\to \infty }{\lim}\frac{1}{\left(2N+1\right)F_{s}}\sum_{n=-N}^{N}\sum_{m=-\infty }^{\infty }R_{x}\left(t_{n},\tau_{m}\right)\exp\left(-j2\pi n\frac{\alpha }{F_{s}}\right)\exp\left(-j2\pi m\frac{f}{F_{s}}\right)$$

In the case of the second-order cyclostationary signal, SC is continuous at spectral frequency f and discrete at cyclic frequency α. It can be rewritten as follows:

(7) $$S_{x}\left(\alpha ,f\right)=\{\begin{array}{cc} S_{x}^{k}\left(f\right), & \alpha =k/T\\ 0, & \alpha \neq k/T \end{array}$$

where $S_{x}^{k}\left(f\right),k=0,\pm 1,\pm 2,\ldots$ are cyclic spectra.

The alignment composed of non-zero values at a certain cyclic frequency α demonstrates the existence of a sinusoidal modulation in the signal, which also suggests the presence of fault signature during the fault diagnosis process. Estimators of SC analysis, such as averaged cyclic periodogram, cyclic modulation spectrum, and fast estimator of SC, are proposed and discussed, contributing to the practical applications of SC analysis.

## 3. Cyclostationary Analysis Based on Correntropy Function

### 3.1. Fundamental of CCE Function and CCE Spectrum

Similar to formulations of the cyclostationary process of the first and second order, we can obtain the basic expression of the CCE. Let $V_{x}(t,\tau )$ denote the correntropy function for a stochastic process x(t) that exhibits hidden periodicities in its structure and for which the time shift is τ. Assume the correntropy function is periodic with $T_{0}$, then:

(8) $$V_{x}(t+T_{0},\tau )=V_{x}(t,\tau ).$$

Furthermore, represent $V_{x}(t,\tau )$ by Fourier series, as shown as Equation (9):

(9) $$V_{x}(t,\tau )=\sum_{\alpha }V_{x}^{\alpha }(\tau )e^{j2\pi \alpha t}$$

where α=n/T, n∈Z is taken as the cyclic frequency associated with the fault characteristic.

Let us define the CCE function for x(t) as Fourier coefficients $V_{x}^{\alpha }(\tau )$, computed by:

(10) $$V_{x}^{\alpha }=\frac{1}{T_{0}}\int_{-T_{0}/2}^{T_{0}/2}V_{x}(t,\tau )e^{-j2\pi \alpha t}dt.$$

According to those formulations, they are functions of time lag τ and are indexed by the cyclic frequency α. Note that for α=0, the CCE function returns to the conventional correntropy function.

Combined with Equation (2), the CCE function can be rewritten as [23]:

(11) $$V_{x}^{\alpha }=\underset{T\to \infty }{\lim}\frac{1}{T}\int_{-T/2}^{T/2}\kappa_{\sigma }(x(t),x(t+\tau ))e^{-j2\pi \alpha t}dt.$$

The CCE function has proved to be very useful in signal processing, although its applications are limited to communication engineering. It is often more effective and natural to transform the structure of a cyclostationary signal in the frequency domain. Note that the CCE is a function with variables t and τ, its frequency domain is a two-dimensional (2D) Fourier transform with two frequency variables α and f, and CCE spectrum (CCES) is defined as a Fourier transform of CCE as follows:

(12) $$S_{x}^{\alpha }(f)=\int_{-\infty }^{+\infty }V_{x}^{\alpha }(\tau )e^{-j2\pi f\tau }d\tau .$$

$S_{x}^{\alpha }(f)$ displays the power distribution of the cyclostationary signal with respect to both the spectral frequency f, which is associated with the system resonance frequency, and the cyclic frequency α, which is also known as the fault frequency.

### 3.2. Kernel Size Selection of the CCE Function

The Gaussian variance, also known as kernel size, is a free parameter which must be chosen according to certain applications. A well-tuned kernel size can minimize the effect of the noise interruption. Therefore, the diagnosis performance of cyclic correntropy is largely determined by the kernel size. However, in previous studies related to cyclic correntropy, the selection method of kernel size was not addressed in detail [21,22,23] and kernel size mainly was decided by multiple trials. Thus, it is necessary to find a solution for the kernel size selection.

The correntropy function is an extended work of the information theoretic learning (ITL) theory [31,32]. Thanks to the work of Robert et al. [33], the link between the inner product in a kernel feature and the ITL cost function was discovered. Robert et al. claimed that the ITL cost functions, when estimated by the Parzen method, can be expressed in terms of inner products in a kernel feature space defined by a Mercer kernel. This link offers a criterion for the selection of the Mercer kernel size based on density estimation.

Among those kernel size estimation methods [34,35,36], Silverman’s rule is a classical method because of its low computation cost and robust performance [37]. Therefore, this paper selects the optimal parameter according to Silverman’ rule, one of the most widely used kernel estimation methods. Silverman’s rule is shown here as Equation (13):

(13) $$\sigma =0.9AN^{-1/5}$$

where N is the data length. A stands for the minimum of the empirical standard deviation of data and the data interquartile range scaled by 1.34.

The influence of the kernel size on the bearing fault diagnosis result is discussed more in greater depth in Section 4 with detailed examples.

### 3.3. Estimation of CCES

Based on the introduction of above two sections, the estimation of CCES is shown as follows:

Step 1. Denote the input signal x[n], with signal length n. Then divide the input signal into L blocks, with each block of N samples.

Step 2. Calculate the kernel size $\sigma_{l}$ of each block with Silverman’s rule, l=0,1,2,…,L−1.

Step 3. Calculate the average of the correntropy function for each block l=0,1,2,…,L−1:

(14) $$M_{l}=\frac{1}{N^{2}}\sum_{\tau_{n}=0}^{N-1}\sum_{n=0}^{N-1}G_{\sigma_{l}}(x_{l}[n],x_{l}[n+\tau_{n}]).$$

Step 4. Calculate the following formulation for each block of size N with α[n]=n/N, n=0,1,2,…N−1, l=0,1,2,…,L−1:

(15) $$V_{l}^{\alpha_{n}}[\tau_{n}]=\sum_{n=0}^{N-1}\left\{[G_{\sigma_{l}}(x_{l}[n],x_{l}[n+\tau_{n}])-M_{l}]e^{-j2\pi \alpha_{n}n}\right\}.$$

Step 5. Calculate the mean value $V_{l}^{\alpha_{n}}[\tau ]$ of all L blocks:

(16) $$V^{\alpha_{n}}[\tau_{n}]=\frac{1}{L}\sum_{l=0}^{L-1}V_{l}^{\alpha_{n}}[\tau_{n}].$$

Step 6. Calculate the discrete Fourier transform for each $\tau_{n}$:

(17) $$T^{\alpha_{n}}[k]=|\frac{1}{N}\sum_{\tau_{n}=0}^{N-1}V^{\alpha_{n}}[\tau_{n}]e^{-j\frac{2\pi }{N}k\tau_{n}}|.$$

Step 7. To clearly observe the fault frequency, project the CCES into the cyclic frequency domain and obtain the cyclic domain profile.

## 4. Validation of CCES on Bearing Fault Diagnosis

### 4.1. Estimation of CCES

The following example aims to illustrate the failure of the Fast Kurtogram in the presence of impulsive noise. Firstly, the simulated bearing fault signal is modeled as a single-degree-of-freedom system according to Reference [38]:

(18) $$y(k)=\sum_{r}\exp(-\alpha *(k-r*F_{s}/f_{m}-\tau_{r})/F_{s})*\sin(2\pi f*(k-r*F_{s}/f_{m}-\tau_{r})/F_{s})$$

where α is equal to 900; $f_{m}$ is the fault frequency, which is set to 100 Hz; and $F_{s}$ is the sampling frequency, which is set to 10,000 Hz. f is the resonant frequency, which is equal to 1000. $\tau_{r}$ is subject to discrete uniform distribution, and is thus used to simulate the randomness caused by roller slippage.

**Figure 1:**
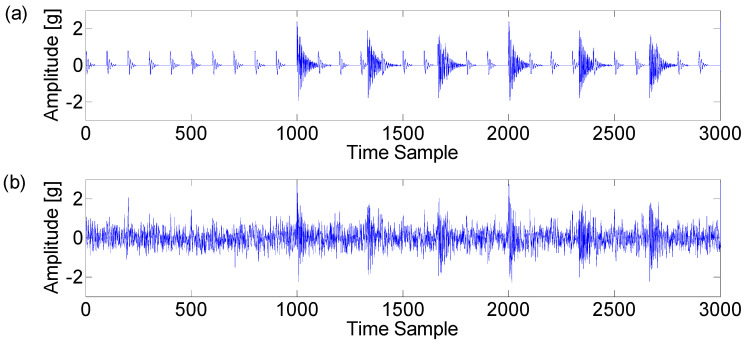
(**a**) Synthetic signal simulating multiple transients produced by faulty bearing and further interrupted by impulsive noise; (**b**) the same signal with additive white Gaussian noise (SNR = −6 dB).

Impulsive noise has a similar behavior to the bearing fault signal. According to the impulsive noise simulation method proposed by Antoni [15], the noise is similarly modeled as a single-degree-of-freedom system with different parameter sets. Typically, α is equal to 300, while $f_{m}$ is set to 30 Hz. The resonant frequency is equal to 3000. Note that the impulsive noise is not composed of a single transient but rather multiple noisy transients in the time domain. The final synthetic signal of length $L=10^{4}$ is displayed in Figure 1. Figure 1a shows that impulsive noise is distributed around sample points 1000, 1334, and so on, whose amplitudes are obviously larger than normal transients of the simulated fault signal. Figure 1b displays the same signal after the addition of white Gaussian noise with signal to noise ratio (SNR) equalling −6 dB.

**Figure 2:**
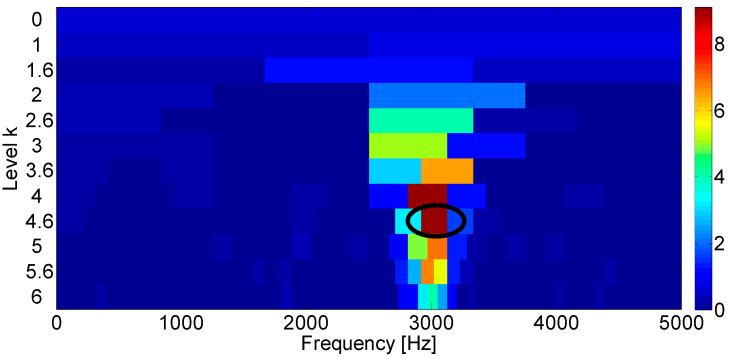
Fast Kurtogram of the simulated bearing fault signal.

**Figure 3:**
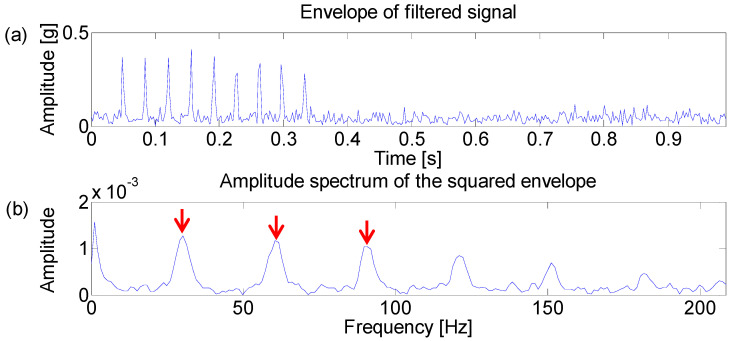
(**a**) Envelope of the filtered signal which maximizes the Kurtogram; (**b**) amplitude spectrum of the squared envelope.

The Fast Kurtogram method [7] is applied to select an optimal band for the square envelope analysis and the corresponding Kurtogram is displayed in the Figure 2. It can be seen that the impulsive noise around 3000 Hz dominates the Kurtogram at level 4.5, while the fault signature is masked in the noise. Furthermore, the frequency band centered at 3020 Hz is obtained by the band filter and the corresponding envelope and amplitude spectrum are displayed in Figure 3. Figure 3b shows that the filtered signal band is actually the impulsive noise component, whose frequency is set to be 30 Hz.

**Figure 4:**
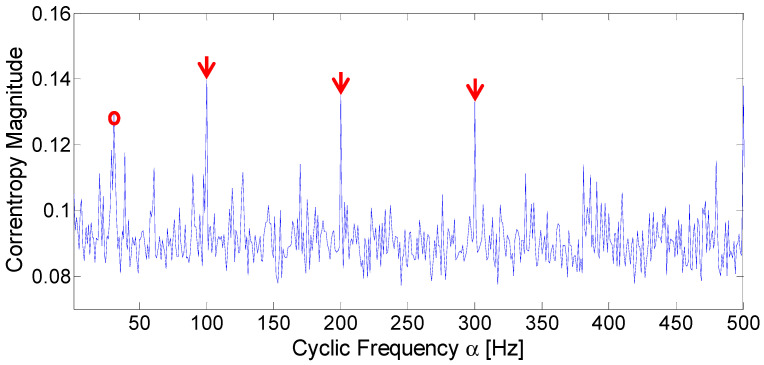
Cyclic domain profile of the CCES for simulated signal.

Then, the simulated signal is further analyzed with the CCES proposed in Section 3.3. To observe the fault frequency in greater detail, the CCES is projected onto the cyclic domain. According to Silverman’s rule, σ is equal to 0.107. The cyclic domain profile when σ is equal to 0.107 is displayed in Figure 4. It can be seen from the figure that the fault frequency is easily identified, marked with red arrows. The spectrum also displays the frequency component of 30 Hz, marked with a red circle. Compared to the fault frequency at 100 Hz and its harmonics, the impulsive noise is largely suppressed. Thus, the analysis results of the simulated signal under an impulsive environment demonstrates that the proposed method can detect the fault frequency effectively.

### 4.2. Case Study 1

To further testify the performance of the proposed method, comparison experiments are conducted on real bearing data. The first example applies the dataset from the Western Reserve University (WRU) bearing data center [39]. This dataset is free from impulsive noise and can be used to test the performance of the proposed method in the presence of common background noise. The data of inner race faults numbered 169 are chosen for analysis. According to formulas for bearing fault characteristic frequency [40], the inner race fault frequency is 162.185 Hz.

**Figure 5:**
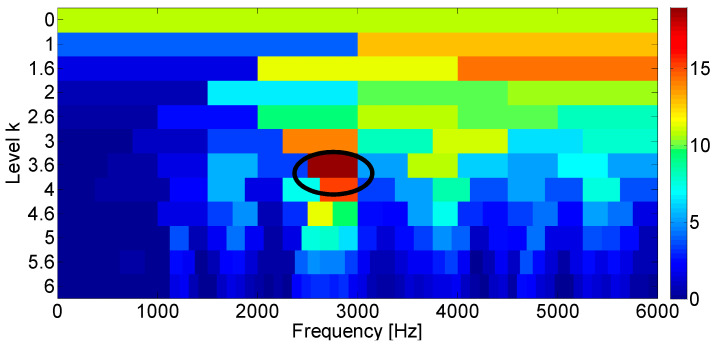
Fast Kurtogram of inner race fault signals numbered 169.

**Figure 6:**
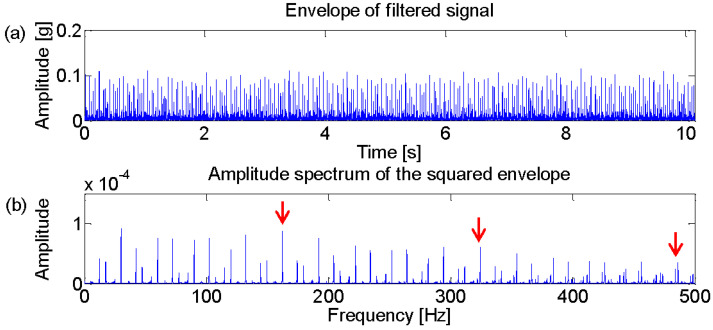
(**a**) Envelope of the filtered signal which maximizes the Kurtogram; (**b**) amplitude spectrum of the squared envelope.

Firstly, the Fast Kurtogram is obtained for the optimal band selection, as shown in Figure 5. It can be seen from the figure that the optimal band is located at level 6 and the frequency center is 2750 Hz, marked with the black circle. This optimal band acquired through the bandpass filter and squared envelope spectrum is displayed in Figure 6. The fault frequency and its harmonics are marked with red arrows. Figure 6b shows that the fault signature is diagnosable but shows some discrete components interrupting the frequency domain.

**Figure 7:**
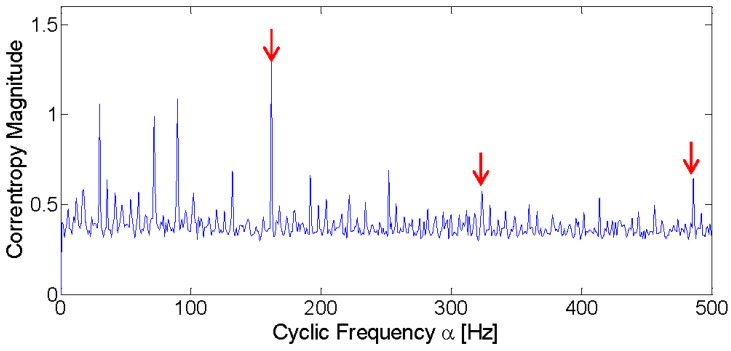
Cyclic domain profile of the CCES for Western Reserve University (WRU) bearing data numbered 169.

Then, with σ set equal to 0.015, the cyclic domain profile of the corresponding signal is displayed in Figure 7. Compared to the squared envelope of the filtered signal based on Fast Kurtogram, the fault frequency and its harmonics are easier to find. The result demonstrates that the proposed method can still obtain a better diagnosis performance compared to the Fast Kurtogram in an environment with common background noise.

### 4.3. Case Study 2

In this case study, industrial railway axle bearing fault data are used for further comparison experiments. One unique component of the industrial railway axle bearing signal is the impulsive background noise. During the train operation on the rail, impulsive force will be produced when the train passes through curves or small gaps between rail joints. This impulsive force will be conducted to the bearing casing through the train wheel and creates an impulsive noise environment. This phenomenon raises new challenges to the bearing fault diagnosis. Focusing on this issue, railway bearing data under impulsive noise interference are acquired to validate the superiority of the proposed method.

**Figure 8:**
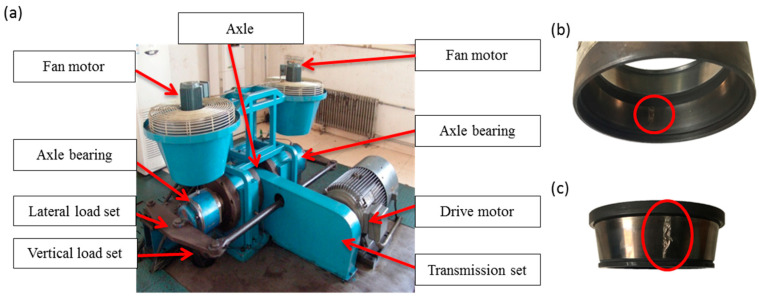
A designed experimental platform and industrial railway axle bearing faults: (**a**) the designed experimental platform; (**b**) the outer race fault; (**c**) the inner race fault.

Our experimental platform for collecting railway axle bearing fault data is shown in Figure 8. Through a transmission set, a variable speed DC motor with a speed up to 1480 r/min is used to drive the rotation of an axle at different speeds. Axle bearings are assembled at the ends of the axle. A lateral load set and a vertical load set are installed to impose practical loads during rail vehicle operation. Note that the lateral load is used to simulate multiple impacts when the train passes curves or small gaps between rail joints. Fan motors are installed to simulate the effect of natural wind in the opposite direction of the vehicle’s momentum. Sensors are mounted at 12 o’clock (directly in the vertical load zone) and 3 o’clock (orthogonal to the vertical load zone) of the bearing casing to acquire vibration data. Two fault bearings are selected from the railway maintenance center and their degradation conditions are shown in Figure 8b,c respectively. The sampling frequency is set to 12,800 Hz. The simulated speed and vertical load are set to 150 km/h and 272 kN, respectively. The lateral load for generating the impulsive noise is 20 kN. According to the transmission ratio of our experimental platform, the inner race fault frequency and the outer race fault frequency are calculated as 164 Hz and 120 Hz, respectively.

**Figure 9:**
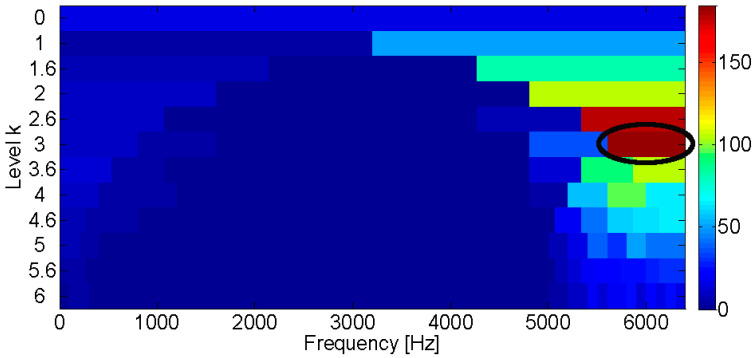
Fast Kurtogram of the axle bearing signal of the inner race fault.

**Figure 10:**
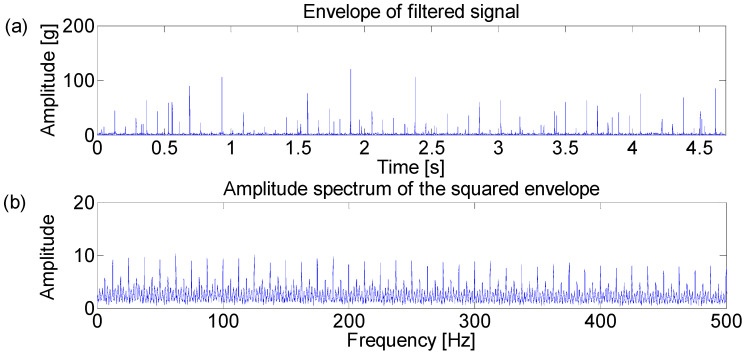
(**a**) Envelope of the filtered signal which maximizes the Kurtogram; (**b**) amplitude spectrum of the squared envelope.

The input data are firstly analyzed with the Fast Kurtogram method and Figure 9 displays the optimal band marked with the black circle. It can be seen from the figure that the optimal band is obtained at level 3, for which the central frequency is 6000 Hz. The associated squared envelope spectrum is further displayed in Figure 10 based on the optimal band. It is found that the Fast Kurtogram fails to find an informative spectral frequency band relevant to the fault signature for further squared envelope spectrum analysis. In Figure 10b, the frequency components are mainly impulsive noise produced by the lateral load. Therefore, it should be noted that the frequency bands associated with the several largest kurtosis values are not enough to establish a correct spectral frequency range for fault diagnosis of the industrial railway axle bearing.

**Figure 11:**
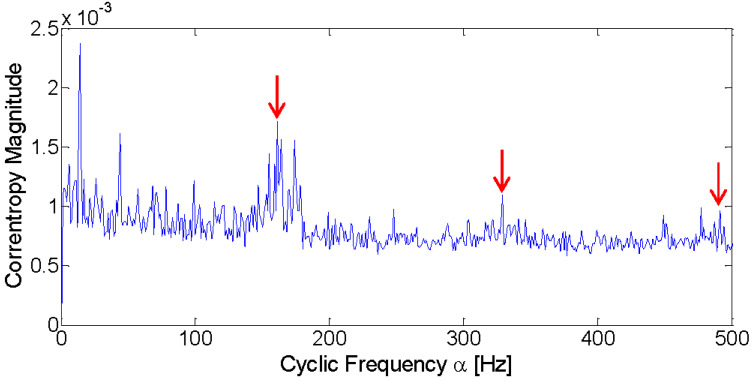
Cyclic domain profile of the CCES for the railway axle bearing signal of the inner race fault.

Then, the CCES is applied to analyze the input data of the inner race fault and the corresponding cyclic domain profile is displayed in Figure 11. According to Silverman’s rule, the σ is set to 4.496 in this experiment. The highlighted frequencies marked with red arrows are 161 Hz, 329 Hz, and 491 Hz. One thing that should be noted is that there is a minor difference between the theoretic fault frequency and the obtained frequency. This is due to random slips between rolling elements and the inner race caused by the large vertical load [41,42]. Furthermore, irregular rotations of the faulty bearing caused by the high speed and vertical load may also have some influence on the fault frequency calculation.

**Figure 12:**
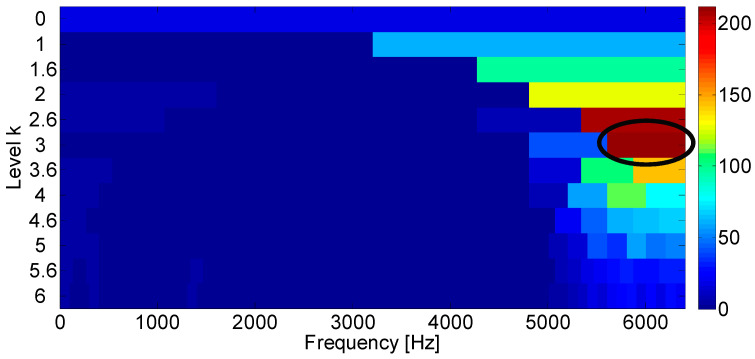
Fast Kurtogram of the axle bearing signal of the outer race fault.

**Figure 13:**
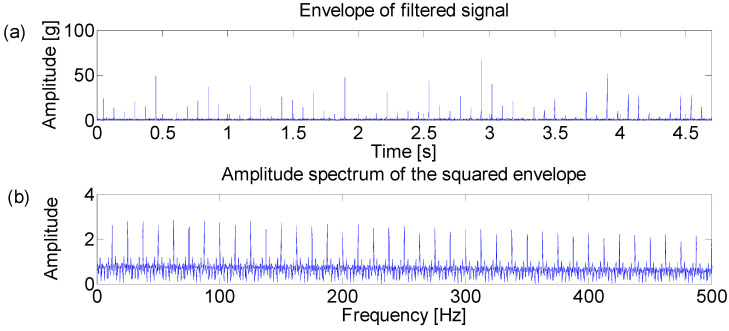
(**a**) Envelope of the filtered signal which maximizes the Kurtogram; (**b**) amplitude spectrum of the squared envelope.

The previous procedure is applied to analyze the axle outer race fault signal and the relevant results are plotted in Figure 12 and Figure 13, respectively. The center frequency of the optimal band is located at level 3 with 6000 Hz. Furthermore, the squared envelope analysis based on the optimal band is displayed in Figure 13. Unfortunately, the fault signature seems to be buried in the impulsive noise again.

**Figure 14:**
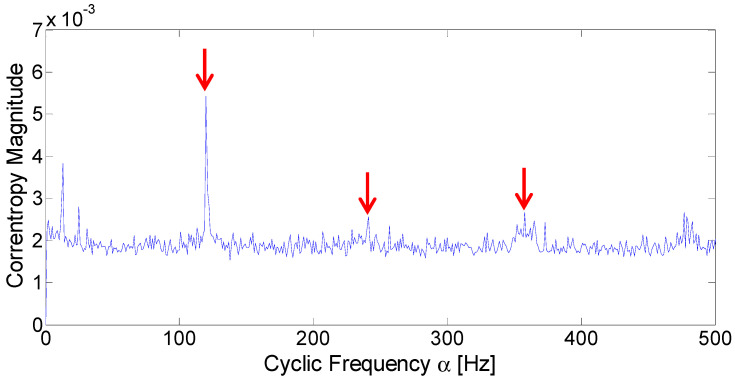
Cyclic domain profile of the CCES for the railway axle bearing signal of the outer race fault.

The application of the CCES method with σ equal to 3.021 and the cyclic domain profile of outer race fault signal is shown in Figure 14. The highlighted frequencies marked with red arrows are 120 Hz, 241 Hz, and 358 Hz, which are almost the same as the theoretic fault frequency and its harmonics.

An interesting point is that the CCES can obtain the cyclic frequency of the fault signature while suppressing the periodic noise, which also is achieved in the simulation signal analysis. Generally speaking, cyclostationary modeling aims to determine all those cyclostationary components in the signal. According to the CCES procedure proposed in Section 3, it is found that this method can easily deal with impulsive noise without any cyclostationarity. When the impulsive noise is cyclostationary, which can also be considered periodic in time domain, the CCES can also detect this component and the cyclic domain profile will show this cyclic frequency in the spectrum plot. However, this frequency component will not dominate the frequency spectrum because this phenomenon only happens when impulsive noise is distributed all along the whole signal and the impulsive amplitude is large enough, which rarely occurs during train operation or in other industries.

### 4.4. Influence of Kernel Size on the Diagnosis Performance

The Gaussian kernel size is a free parameter selected according to certain applications. The variation of kernel size expands its application range, while improper kernel size may become its potential weakness. Previous studies on the kernel size selection of CCE were mainly based on multiple trials, which does not lead to the optimal parameter for the Gaussian kernel function. In this section, fault diagnosis performance of different kernel size is analyzed to provide an effective mechanism for kernel size selection.

Input data are derived from the above two case studies for comparison, including the WRU data of the inner race fault numbered 169 and the axle bearing data of the outer race fault. The optimal kernel size of each experiment is based on Silverman’ rule. In addition, another random kernel size is used for comparisons.

**Figure 15:**
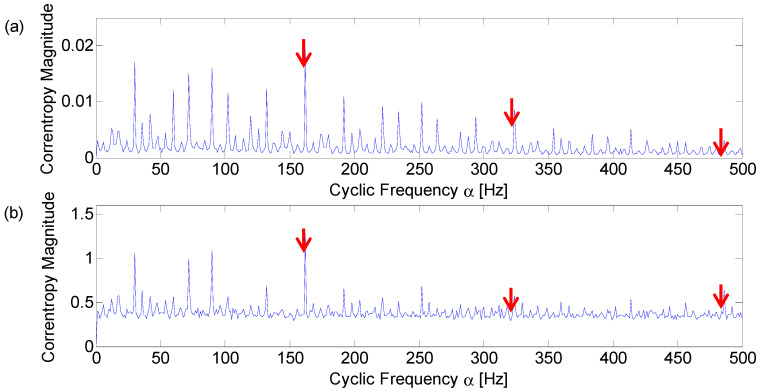
Cyclic domain profile of the CCES for WRU bearing data numbered 169 with different kernel size: (**a**) *σ* = 1; (**b**) *σ* = 0.015.

The random kernel size is set to 1 and the optimal kernel size is 0.015 according to Silverman’s rule. The analysis results of the WRU data numbered 169 are displayed in Figure 15. Both of the two analysis results can identify the fault frequency and its harmonics. However, Figure 15b with the optimal kernel size displays fewer frequency components than Figure 15a with a random kernel size, especially in the frequency band between 100 Hz and 350 Hz. This is because those frequency components which are uncorrelated to the fault signature have been suppressed.

**Figure 16:**
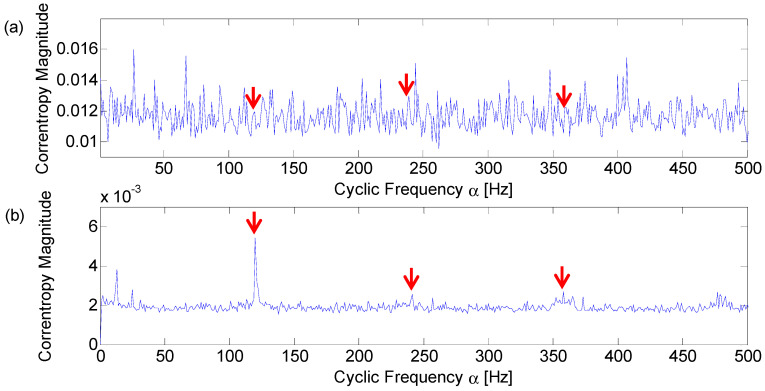
Cyclic domain profile of the CCES for the railway axle bearing signal of the outer race fault with different kernel sizes: (**a**) σ = 0.1; (**b**) σ = 3.021.

Similarly, the random kernel size is set to 0.1 and the optimal kernel size is 3.021 according to Silverman’s rule. The analysis results of the axle bearing signal of the outer race fault are displayed in Figure 16. It can be seen from the Figure 16a that the result with the randomly chosen kernel size cannot locate frequencies corresponding to the fault characteristic. Compared with Figure 16a, the cyclic domain profile with the optimal kernel size exhibits a much better diagnosis performance, as shown in Figure 16b.

The above two analysis results have shown that the kernel size plays an important role in the fault diagnosis through the CCES method, especially when signal components are complex and affected by impulsive noise. The kernel optimization method based on Silverman’s rule is highly efficient and accurate during the fault diagnosis. Thus, CCES can be regarded as a self-adaptation method which may also be applied other objects in mechanical fields.

## 5. Conclusions

In this paper, we have investigated an impulsive noise suppression method for bearing fault diagnosis based on the CCE function. Silverman’s rule was used to obtain an optimal kernel size for CCE input. This adjustable parameter provides an effective mechanism to eliminate the negative effect of impulsive noise. Then, a simulated signal and two real cases were studied to validate the performance of the proposed method. The experiment results show that the proposed method has a good diagnosis performance, especially in the presence of impulsive noise. Furthermore, a powerful frequency band selection method named Fast Kurtogram was applied to analyze the same data for comparison. It was found that the fault diagnosis method based on CCE function outperforms the Fast Kurtogram method.

Further research should mainly focus on the combination of CCE and mode identification methods. Typically, frequency domain features of CCES can be extracted to reflect different fault characteristics. Moreover, considering the computation time of cyclic methodology, a simplified and fast computation method should be developed in subsequent research works. Note that this new method can generate cyclostationary signatures for bearing fault diagnosis. Thus, it should be helpful in the diagnosis of other machinery, such as gears and blades.
